# Clinical and Genetic Features of Autosomal Recessive Bestrophinopathy: A Case Series from a Vietnamese Cohort

**DOI:** 10.3390/biomedicines13071625

**Published:** 2025-07-02

**Authors:** Trang Thi Thu Nguyen, Van Khanh Tran, Ngoc Lan Nguyen, Nguyen Van Huy, Thinh Huy Tran, Le Thi Phuong, Phan Long Nguyen, Thuy Thu Nguyen, Tran Thi Quynh Trang, Do Thanh Huong, Ngo Thi Thu Huong, Trong Van Pham, Quoc Tung Mai

**Affiliations:** 1 Hanoi Medical University, 1st Ton That Tung Street, Hanoi 11521, Vietnam; trangntt@tbump.edu.vn (T.T.T.N.); dothanhhuong@hmu.edu.vn (D.T.H.); thuhuong@hmu.edu.vn (N.T.T.H.); 2 Center for Gene and Protein Research, Hanoi Medical University, 1st Ton That Tung Street, Hanoi 11521, Vietnam; tranvankhanh@hmu.edu.vn (V.K.T.); nguyenngoclan@hmu.edu.vn (N.L.N.); phuongle@hmu.edu.vn (L.T.P.); phanlongnguyen1998@gmail.com (P.L.N.); nguyenthuthuy@hmu.edu.vn (T.T.N.); tranthiquynhtrang@hmu.edu.vn (T.T.Q.T.); 3 National Institute of Ophthalmology, 85 Ba Trieu Street, Hanoi 11614, Vietnam; huynhannhi@yahoo.com; 4 Biochemistry Department, Hanoi Medical University, 1st Ton That Tung Street, Hanoi 11521, Vietnam; tranhuythinh@hmu.edu.vn; 5 Department of Ophthalmology and Optometry, Hanoi Medical University, 1st Ton That Tung Street, Hanoi 11521, Vietnam; phamtrongvan@hmu.edu.vn

**Keywords:** autosomal recessive bestrophinopathy (ARB), wide-field fundus photography, optical coherence tomography (OTC), vitelliform lesions, subretinal deposits, diffuse macular hyperfluorescence, *BEST1* variant, Vietnamese

## Abstract

**Objectives**: This study aims to describe the clinical features and genetic findings of nine Vietnamese patients with autosomal recessive bestrophinopathy. **Methods**: This retrospective and cross-sectional study included individuals diagnosed with autosomal recessive bestrophinopathy at the Eye Clinic, Vietnam National Geriatric Hospital between May 2024 and April 2025. The patients underwent a visual acuity assessment, retinal multimodal imaging, and molecular testing through *BEST1* gene sequencing. **Results**: Nine patients from seven unrelated families were included. The mean age was 38.6 years (range: 14.1–79.6). Visual acuity ranged from 20/20 to 20/125. All patients showed vitelliform lesions, subretinal deposits, and both intraretinal and subretinal fluid. Other main features included diffuse macular hyperfluorescence and hyperopia. Less common clinical features encompassed glaucoma, retinoschisis, outer retinal thinning, serous retinal detachment, retinal thickening, and thinning of the retinal pigment epithelium. Compound heterozygous or homozygous variants were detected in all patients. Among the five identified *BEST1* variants, the most frequent were p.(A195V) and p.(R200*). One novel variant, p.(K289*), was detected. **Conclusions**: The main clinical retinal features of nine Vietnamese patients with autosomal recessive bestrophinopathy included vitelliform lesions, subretinal deposits, retinal fluid, and diffuse macular hyperfluorescence. The most common variants were p.(A195V) and p.(R200*). Additionally, the identification of various compound heterozygotes and a novel *BEST1* variant expands the mutation spectrum of the disease.

## 1. Introduction

Bestrophinopathies refer to a group of unique inherited retinal dystrophies that primarily impact the macular region, which is responsible for central high-acuity vision, and mainly affect the retinal pigment epithelium (RPE)—a single layer of cells that supports and interacts with the light-sensitive retina [1]. These conditions are associated with mutations in the *BEST1* gene (OMIM * 607854), located on the long arm of chromosome 11 (11q12). The gene comprises 11 exons and encodes bestrophin-1, a 68 kDa protein made up of 585 amino acids [2]. Bestrophin-1 is mainly localized in the basolateral plasma membrane of the RPE and functions as a calcium-activated chloride channel (CaCC) [3,4]. Human bestrophin-1 (hBEST1) [5] forms a pentameric structure which is shown in *Klebsiella pneumoniae* (KpBEST) [6] and chickens (cBEST1) [7]. Bestrophin-1 plays a major role in ocular physiology by regulating the transport of chloride and other monovalent anions across cell membranes in response to intracellular calcium fluctuations and to maintain ionic homeostasis [8,9]. Additionally, CaCC activity is believed to contribute critically to the generation of visual responses to light exposure [10]. Pathogenic variants in *BEST1* can lead to a spectrum of bestrophinopathies, including best vitelliform macular dystrophy (BVMD, OMIM # 153700) [11], adult-onset vitelliform macular degeneration (AVMD, OMIM # 179605) [12], autosomal dominant vitreoretinochoroidopathy (ADVIRC, OMIM # 193220) [13], autosomal recessive bestrophinopathy (ARB, OMIM # 611809) [14], and retinitis pigmentosa (RP, OMIM # 613194) [15].

Autosomal recessive bestrophinopathy (ARB) is a rare inherited retinal disorder first described by Burgess et al. in 2008 [14]. ARB accounted for 1.9% of inherited retinal diseases in an Egyptian cohort [16]. It is characterized by multiple diffuse subretinal deposits that exhibit hyperfluorescence on fundus autofluorescence imaging [17]. Electrophysiological testing typically reveals a significant reduction in the light rise on electrooculography (EOG) [17]. Based on fundus appearance and electrophysiological findings, ARB can be categorized into several clinical phenotypes, including single or confluent yellow lesions at the posterior pole and midperiphery; small fleck-like lesions extending to the far periphery; a widespread continuous lesion with sharply demarcated peripheral borders; and single or multifocal vitelliform macular dystrophy-like lesions [18]. Patients with ARB often present with blurred vision, visual acuity ranging from 20/200 to 20/25, hyperopia, altered colour perception, a short axial length, and glaucoma [19]. Symptom onset typically occurs within the first two decades of life, although later onset up to the fifth decade has also been reported [20]. The estimated incidence of ARB is less than 1 in 1,000,000 individuals [17,19,21]. Diagnosing ARB can be difficult due to the wide variability in clinical presentations, even among siblings or between a patient’s two eyes [18,22]. Comprehensive retinal imaging techniques—such as macular and wide-angle fundus photography, fundus autofluorescence (FAF), and optical coherence tomography (OCT)—are essential for accurately defining the clinical features of ARB and guiding targeted molecular genetic testing to confirm the specific subtype [18,23].

To date, more than 300 variants have been identified in both alleles of the *BEST1* gene, with only about 40 compound heterozygous and homozygous pathogenic variants known to cause ARB [21,24,25,26]. Approximately 50% of the variants associated with ARB are located between amino acid residues 312 and 325 [14,27]. The most common variants vary among different populations. For example, p.R141H is the most common variant reported in European families [28], while p.R255W and p.A195V are more common in individuals of Chinese descent [29,30,31], and p.A195V is the predominant variant in Korean patients [23]. Sanger sequencing [14], next-generation sequencing [32], and third-generation sequencing [33] have been used to identify variants in the *BEST1* gene in patients with ARB.

To our knowledge, no studies on ARB in Vietnamese patients have been published to date. In this study, we conducted a retrospective, cross-sectional analysis of nine Vietnamese patients with ARB, focusing on their clinical features and genetic variants.

## 2. Materials and Methods

### 2.1. Individuals

This study included nine patients (six males and three females) from seven unrelated families (Table 1). Participants were eligible for inclusion if they were of Vietnamese descent and exhibited clinical features suggestive of autosomal ARB. Inclusion criteria included: (1) the presence of multifocal subretinal fluid or vitelliform lesions observed on fundus examination and confirmed by OCT, (2) abnormal FAF, (3) genetic confirmation of biallelic pathogenic or likely pathogenic variants in the *BEST1* gene, (4) age ≥ 6 years, to ensure reliable cooperation for imaging and functional assessments, (5) the ability and willingness to undergo a comprehensive ophthalmic evaluation, including best-corrected visual acuity testing, refraction, fundus examination and photography, OCT, and FAF imaging. Individuals were excluded if they harboured only monoallelic *BEST1* variants without clinical features consistent with ARB, as these may represent carriers or individuals with dominantly inherited forms, such as BVMD. Additional exclusion criteria included: (1) the presence of other co-existing inherited retinal dystrophies confirmed by clinical history or genetic testing (e.g., *ABCA4*, *RPGR*, and *CRB1*), (2) significant ocular comorbidities that could confound the retinal phenotype, (3) media opacities, such as dense cataracts or corneal opacities, that precluded the acquisition of high-quality retinal imaging. P2 and P3 are siblings, as are P8 and P9. All patients had a documented ophthalmic history prior to admission to our department (Table 1). Hyperopia was observed in 77.8% of patients (7/9), and glaucoma was present in 33.3% (3/9). Patient P1 was diagnosed with chronic central serous choroidopathy with choroidal neovascularization (CNV) and open-angle glaucoma. The patient underwent trabeculectomy for glaucoma and received intravitreal bevacizumab injections, but the symptoms did not improve. Patient P3 presented with choroidal neovascularization and polypoidal choroidal vas-culopathy and received intravitreal bevacizumab injections. Two patients (P5 and P8) were treated only with artificial tears.

### 2.2. Fundus Examination

Fundus examination with multimodal imaging investigation was conducted at the Eye Clinic, Vietnam National Geriatric Hospital. Wide-field fundus photography and FAF were obtained using the Optos California system (Optos plc, Dunfermline, Scotland), while fluorescein angiography (FFA) was performed with the Kowa VX-10i (Kowa Company Ltd., Shizuoka, Japan). OCT images were acquired using the Optopol RENO NX 130 (Optopol Technology Ltd., Zawiercie, Poland).

### 2.3. Variant Screening of the BEST1 Gene

Total genomic DNA was extracted from patients’ whole blood using the QIAamp DNA Mini Kit (QIAGEN, Hilden, Germany). Polymerase chain reaction (PCR) was used to amplify the 10 coding exons of the *BEST1* gene with specific primer pairs (Table 2). PCR amplification was performed using the Mastercyles X50S-PCR Thermocycler (Eppendorf, Hamburg, Germany) as follows: an initial denaturation at 95 °C for 5 min, 35 cycles of 95 °C for 30 s, 60 °C for 30 s, and 72 °C for 30 s, and an extension at 72 °C for 5 min. Sanger sequencing was performed with the BigDye Terminator v3.1 Cycle Sequencing Kit (Applied Biosystems, Vilnius, Lithuania) on an ABI 3500 Genetic Analyzer (Applied Biosystems, Foster City, CA, USA). Sanger sequencing was also conducted on healthy individuals and on family members of patient P2. The variants were determined by aligning sequencing data with the reference sequences NG_009033 and NM_004183 of the *BEST1* gene using the CLC Main Workbench software v6.0.1 (Qiagen, Venlo, The Netherlands).

*BEST1* variants were cross-checked with the Genome Aggregation Database (gnomAD v4.1.0) [34], dbSNP157 [35], and ClinVar [36]. The effects of the variants on protein function were predicted using MutationTaster2021 [37], Combined Annotation Dependent Depletion (CADD) v1.7 [38], Sorting Intolerant From Tolerant (SIFT) [39], and Polymorphism Phenotyping v2 (PolyPhen_2) [40]. The pathogenicity of the variants was assessed according to the American College of Medical Genetics and Genomics (ACMG) guidelines [41].

## 3. Results

### 3.1. Clinical Findings

**Figure 1:**
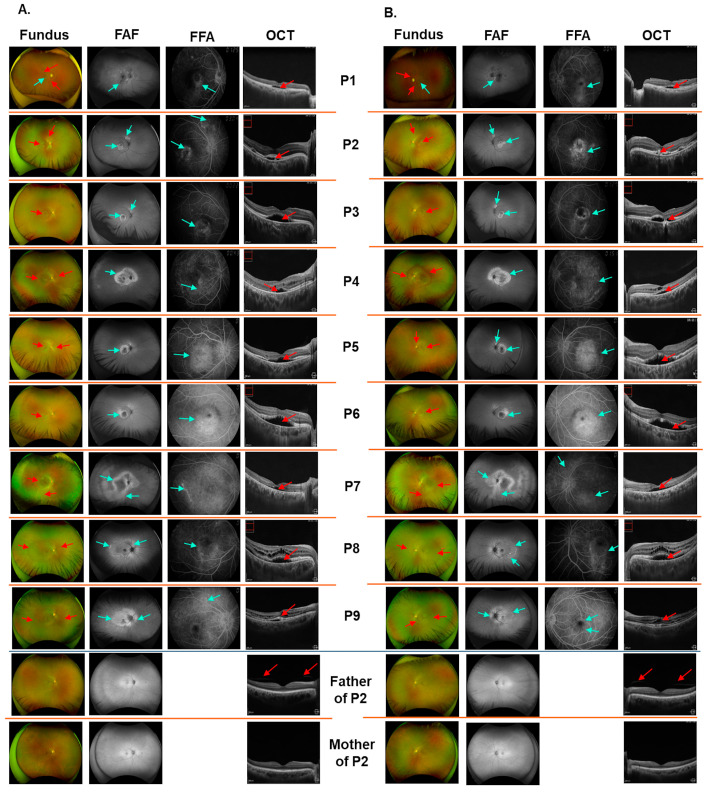
Multimodal imaging of right eye (**A**) and left eye (**B**) of nine patients with autosomal recessive bestrophinopathy and the unaffected parents of P2. FFA, fundus fluorescein angiography; FAF, fundus autofluorescence; OCT, optical coherence tomography. Wide-angle fundus colour images showed a vitelliform under the macula (fundus images). OCT images indicated retinal thickening and fluid, and disruption of photoreceptor elongation and epiretinal membrane in patient P1; subretinal fluid and subretinal deposition of lipofuscin in patients P2 and P3; subretinal fluid and diffuse deposits, retinoschisis, and RPE thinning in patient P4; subretinal fluid and deposits, outer retinal thinning, and photoreceptor disruption and elongation in patients P5 and P6; subretinal fluid and outer retinal thinning in patient P7; and subretinal fluid and deposits, retinoschisis, and photoreceptor elongation and disruption in patients P8 and P9. The unaffected parents of P2 exhibited normal colour fundus, FFA, and OCT images. The OCT images revealed a posterior vitreous detachment in the P2’s father, which is commonly observed at his age (>60-year-old).

A total of nine patients were included in this study. All patients were referred to the Eye Clinic, Vietnam National Geriatric Hospital and diagnosed with ARB, which was confirmed through genetic testing. The mean age was 38.6 years (range: 14.1–79.6). Visual acuity ranged from 20/20 to 20/125 (Table 3). All patients showed vitelliform lesions, subretinal deposits, and both intraretinal and subretinal fluid (Table 3 and Figure 1). Diffuse macular hyperfluorescence on fundus autofluorescence imaging was observed in 88.9% of patients (8/9). Retinoschisis and outer retinal thinning were present in 33.3% (3/9), while retinal thickening and thinning of the retinal pigment epithelium were noted in 11.1% (1/9) (Table 3 and Figure 1). Foci of deposits were detected around the optic disc in three patients (P1, P2, and P3), around the optic discs in four patients (P4, P7, P8, and P9), and near the vascular arcade and above the optic disc in two patients (P5 and P6) (Figure 1).

The oldest patient, P1 (79.6-year-old), showed a severe retinal phenotype, such as diffuse macular hyperfluorescence, serous retinal detachment, mixed areas of hyper- and hypo-autofluorescence due to RPE alterations, retinal thickening and fluid accumulation, and disruption of the epiretinal membrane (Table 3 and Figure 1). Two siblings, P2 and P3, presented with similar retinal features; however, P3 had poorer visual acuity than P2 (BCVA of P3: OD 20/100; OS 20/125, compared with BCVA of P2: OD 20/40; OS 20/50). Patient P4 showed evidence of retinoschisis and RPE thinning. Similarly, siblings P8 and P9 had comparable retinal phenotypes but differed in visual acuity. Patient P8 showed a slight reduction in BCVA (OD 20/25; OS 20/32), while patient P9 had normal vision (20/20 in both eyes).

### 3.2. Analyses of BEST1 Variants

**Figure 2:**
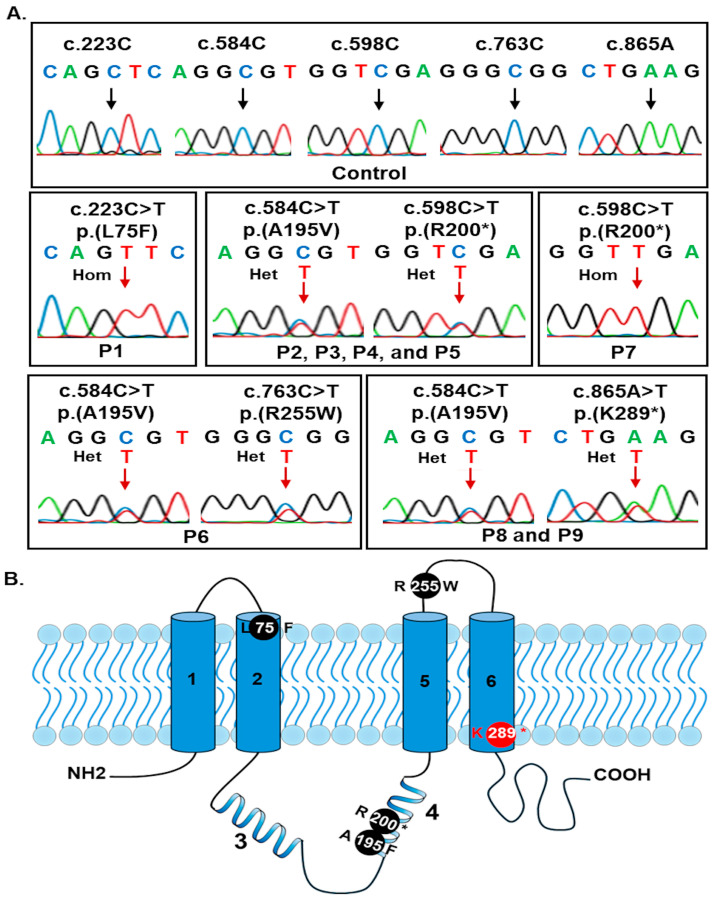
Sanger sequencing of the *BEST1* variants identified in nine patients (**A**) and the location of mutant residues in the predicted topology of human bestrophin-1, adapted to Milenkovic et al. [42] (**B**). In Figure 2A, the black arrows represent normal nucleotides, the red arrows indicate mutant nucleotides; Hom, homozygous; Het, heterozygous. In (**B**), the known mutant residues are marked in black, while the novel mutant residue is marked in red. Hom, homozygous; Het, heterozygous.

Five *BEST1* variants were identified in nine patients, including three missense variants and two nonsense variants (Figure 2A). Of these, four variants—c.223C>T p.(L75F), c.584C>T p.(A195V), c.598C>T p.(R200*), and c.763C>T p.(R255W)—had been previously reported, while one variant, c.865A>T p.(K289*), was novel (Table 4 and Figure 2). Two patients harboured homozygous variants and seven patients carried compound heterozygous variants. The most frequent variants were c.584C>T p.(A195V), present in 66.7% of patients, and c.598C>T p.(R200*), present in 55.6%. The remaining three variants were each found in a single patient.

The c.223C>T p.(L75F) variant was predicted as a damaging variant in MutationTaster2021 or CADD tools, but benign according to PolyPhen_2 and SIFT (Table 4). It was classified as a likely pathogenic variant in the ClinVar database (ID 3767340). The other four variants were consistently predicted as damaging or deleterious by in silico tools (Table 4). All five variants were classified as either pathogenic or likely pathogenic according to ACMG guidelines (Table 4).

**Figure 3:**
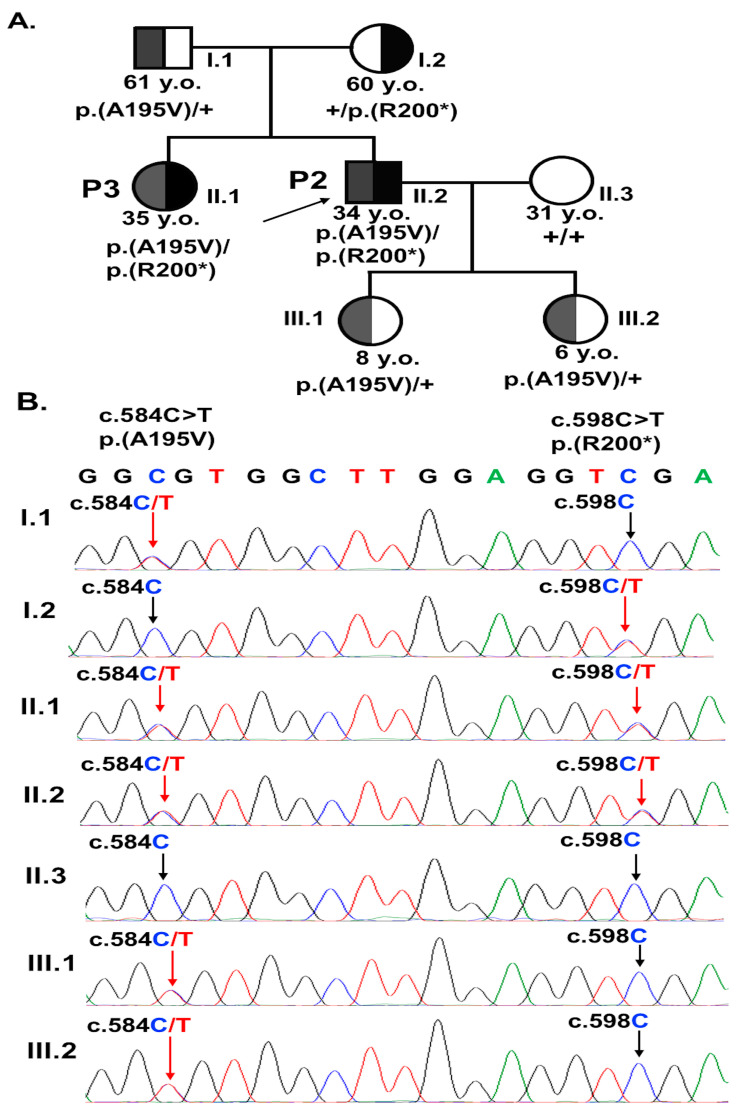
Pedigree (**A**) and Sanger sequencing electropherograms (**B**) of the two variants, c.584C>T p.(A195V) and c.598C>T p.(R200*), located on exon 5 of *BEST1* gene in the family of P2 and P3. Shaded symbol + represent wild-type allele. The patients P2 and P3 harboured compound heterozygous variants. The father (I.1) and two daughters of P2 (III.1 and III.2) carry the variant c.584C>T p.(A195V) in the heterozygous state. The mother (I.2) carries the variant c.598C>T p.(R200*) in the heterozygous state. The spouse of P2 has wild-type alleles.

Sanger sequencing revealed that P2 and P3 inherited the missense variant c.584C>T p.(A195V) from their father and the nonsense variant c.598C>T p.(R200*) from their mother (Figure 3). The two daughters of P2 carry c.584C>T p.(A195V) in the heterozygous state (Figure 3). The ocular examinations of the heterozygous carrier parents revealed that they did not exhibit any clinical symptoms of ARB (Figure 1). Hence, individuals with mutations in both copies of the *BEST1* gene display a clinical phenotype characteristic of ARB.

## 4. Discussion

Diagnosing bestrophinopathy is often challenging due to the variability in symptom onset. An accurate diagnosis requires a combination of ophthalmological assessment and genetic analysis. In this study, by integrating clinical features with *BEST1* variant screening, we made a definitive diagnosis of ARB for nine Vietnamese patients. These patients exhibited the typical features of ARB, including vitelliform lesions, subretinal deposits, retinal fluid, and diffuse macular hyperfluorescence. Less common symptoms included hyperopia, glaucoma, retinoschisis, and retinal thinning. Serous retinal detachment, retinal thickening, and thinning of the retinal pigment epithelium were rare, observed in only 11.1% of cases. In contrast, a study by Lou et al. involving 21 Chinese ARB patients reported serous retinal detachment in 20 of 21 patients and angle closure or angle-closure glaucoma in 15 of 21 patients [45]. The mean age of patients in this study was 38.6 years (range: 14.1–79.6), which is older than the average onset age of 6 years typically reported for BVMD [8,46]. Visual acuity ranged from 20/20 to 20/125, consistent with previously reported findings in patients with ARB [47].

In this study, five pathogenic or likely pathogenic variants were identified, located on exons 3, 5, and 7—regions outside the typical BMVD hotspots [48]. These findings support the notion of a distinct *BEST1* variant spectrum between ARB and BMVD [27,48]. It has been noted that in individuals with recessive bestrophinopathy, it typically occurs alongside other pathogenic variants which often occur in a compound heterozygous form alongside other variants [14,27,44,49,50]. Our results are consistent with this pattern, as seven out of nine patients in our cohort carried compound heterozygous variants.

The first missense variant, c.223C>T p.(L75F), was identified in patient P1, a 79-year-old male. Leucine 75 is located in the second transmembrane domain (Figure 2B), within the bestrophin channel neck region [51], which plays a key role in channel gating. Mutations in this region can disrupt the pore structure and impair channel function [52]. The L75F substitution may interfere with the activity of the calcium-activated chloride channel at the RPE basolateral membrane, negatively affecting the function of wild-type bestrophin [43]. A homozygous carrier is likely to exhibit a more severe phenotype due to haploinsufficiency [43]. In our study, patient P1’s severe retinal manifestations support this hypothesis. He presented with diffuse macular hyperfluorescence, serous retinal detachment, chronic central serous chorioretinopathy with CNV, and open-angle glaucoma. He underwent trabeculectomy and received intravitreal bevacizumab injections. Interestingly, individuals heterozygous for the c.223C>T p.(L75F) variant have also been reported to present with BVMD [43]. Therefore, genetic screening of patient P1’s family members for the c.223C>T p.(L75F) variant is recommended to identify carriers and provide appropriate genetic counselling, prognosis, and early intervention.

The second missense variant, c.584C>T p.(A195V), is one of the most commonly reported variants associated with BVMD, ARB, and MVD [44,45,48,53]. In our study, this variant was identified in six out of nine patients. Alanine 195 is located within bestrophin’s cytoplasmic loop (Figure 2B), a region where pathogenic variants are often linked to reduced mRNA or protein expression and typically exhibit a recessive mode of inheritance [50,52]. The third missense variant, c.763C>T p.(R255W), is the most common mutation in the Chinese population, with an allele frequency of 52.9% [45]. Arginine 255 is positioned at the end of the third transmembrane domain (Figure 2B), which is also part of the bestrophin neck region [52]. This variant may reduce ion channel permeability, potentially altering the protein’s structure and function [43,52]. In this study, we identified a compound heterozygous missense variant combination—c.584C>T p.(A195V) and c.763C>T p.(R255W)—in a 48-year-old female (P6) presenting with chronic central serous chorioretinopathy, open-angle glaucoma, diffuse macular hyperautofluorescence, RPE alterations, subretinal fluid and deposits, outer retinal thinning, and photoreceptor disruption and elongation. This compound heterozygous variant has been reported in previous studies: in a 25-year-old Chinese patient [53], and in a 21-year-old male and a 42-year-old female from China [45]. The 25-year-old patient exhibited pseudohypopyon in the right eye and a vitelliform lesion in the left eye [53]. The 21-year-old male and 42-year-old female showed similar features such as cystoid macular edema, retinal detachment, elongated photoreceptor outer segments, hyperreflective subretinal deposits, RPE atrophy, and hyperfluorescent spots. However, the 21-year-old male also had peripheral vascular leakage and vessel dilation, while the 42-year-old female demonstrated increased vascular permeability [45]. Patient P6 showed more severe clinical manifestations than previously reported individuals with the same mutations. This could be attributed to P6’s older age, asymmetric retinal degeneration [54], stochastic variability [55], or environmental influences such as prolonged light exposure [56,57].

The first nonsense variant, c.598C>T p.(R200*), results from a cytosine-to-thymine substitution at nucleotide position 598, creating a premature stop codon at amino acid position 200. This leads to the loss of the third and fourth transmembrane domains and the cytoplasmic loop in between (Figure 2B) [14,44]. The resulting truncated bestrophin protein cannot properly localize to the membrane or form a fully functional chloride channel complex [58]. In this study, the c.598C>T p.(R200*) variant was identified in five patients, including one (P7) who carried the variant in a homozygous state. Previous studies have reported several homozygous cases of this variant [14,44]. In one report, two siblings with this variant showed a visual acuity of OD: 20/60 and OS: 20/200 to 20/120, along with subretinal deposits, RPE irregularities, early patchy hyperfluorescence, reduced rod and cone full-field electroretinography, and absent EOG light rise [14]. Another case involved a nine-year-old boy with a visual acuity of OD: 20/25 and OS: 20/80, hyperopia, unremarkable electroretinography, and bilateral multiple, partially confluent yellowish lesions at the posterior pole, with round dome-shaped deposits within the RPE and photoreceptor detachment from the RPE [44]. In this study, patient P7 showed an OD: 20/100 and OS: 20/125, diffuse subretinal deposits, foci of deposits around the optic discs, peripheral and inferior retinal changes, widespread macular hyperautofluorescence, subretinal fluid, and outer retinal thinning.

In our study, four patients (P2–P5) harboured a compound heterozygous combination of c.584C>T p.(A195V) and c.598C>T p.(R200*). These patients presented with macular lesions, vitelliform-like subretinal deposits, and subretinal fluid of varying severity, which may be influenced by age or individual factors. To our knowledge, this is the first study to report the compound heterozygous combination of c.584C>T p.(A195V) and c.598C>T p.(R200*) in ARB patients. The second nonsense variant, c.865A>T (p.K289*), is a novel mutation that has not been previously reported in any databases or the literature. This mutation is located near the end of the fourth transmembrane domain (Figure 2B) and results in a truncated protein consisting of only 288 amino acids. This leads to the loss of the C-terminal region from residue 289 onward, which is essential for calcium ion (Ca^2+^) binding in the RPE [59]. Without this C-terminal domain, the protein cannot bind Ca^2+^ ions effectively, resulting in a severe reduction or complete loss of chloride (Cl^−^) ion current [59].

This study has several limitations. First, the sample size was relatively small (*n* = 9), which may limit the generalizability of the findings to the broader Vietnamese population or ARB patients of different ethnic backgrounds. However, given the rarity of ARB, particularly in Southeast Asian populations, this case series contributes valuable data to the limited existing literature. Second, EOG, a key diagnostic modality for assessing retinal pigment epithelium function in bestrophinopathies, was not performed due to the unavailability of EOG equipment at our institution. This may have limited our ability to characterize the functional phenotype of ARB in these patients comprehensively.

Future studies with larger cohorts and access to complete electrophysiological testing, including EOG, are warranted to elucidate further the clinical spectrum of ARB in Vietnamese and other Asian populations. Additionally, further experimental studies are crucial to understand the functional impact of identified variants.

## 5. Conclusions

Autosomal recessive bestrophinopathy is a rare inherited retinal disease caused by pathogenic variants in the *BEST1* gene. In Vietnamese patients with ARB, the primary retinal phenotypes included vitelliform lesions, subretinal deposits, intraretinal and subretinal fluid, and diffuse macular hyperfluorescence. The most common variants identified were c.584C>T p.(A195V) and c.598C>T p.(R200*). To the best of our knowledge, this is the first report describing ARB in Vietnamese patients. The findings of this study contribute to the understanding of ARB.

## Figures and Tables

**Table 1:** Ophthalmic history of nine patients with autosomal recessive bestrophinopathy.

Patient	Sex	Family History	Ophthalmic History	Treatment History
P1	Male	None	Chronic central serous chorioretinopathy with CNV and open-angle glaucoma.	Trabeculectomy procedure for glaucoma and glaucoma eye drops. Received bevacizumab intravitreal injections without improvement.
P2	Male	Sister (P3)	Maculopathy and hyperopic astigmatism.	None.
P3	Female	Brother (P2)	Choroidal neovascularization, polypoidal choroidal vasculopathy, and hyperopia.	Received bevacizumab intravitreal injections.
P4	Male	None	Maculopathy and open-angle glaucoma.	Glaucoma eye drops.
P5	Male	None	Hyperopic, maculopathy, and exudative retinal detachment.	Artificial tears.
P6	Female	None	Chronic central serous chorioretinopathy, closed-angle glaucoma, and hyperopia.	Laser peripheral iridotomy and glaucoma eye drops.
P7	Male	None	Bullous central serous chorioretinopathy and hyperopia.	
P8	Female	Brother (P9)	Hyperopic astigmatism and macular edema.	Artificial tears.
P9	Male	Sister (P8)	Hyperopic astigmatism and macular edema.	

**Table 2:** The primers used in this study.

Exon	Forward (3′–5′)	Reverse (3′–5′)	Length (bp)
2	GAGAGTTGAGGTCCAGAGCA	GCAGCCTCTCAGTCTGACTT	484
3	GTTTGGGGCTGTACAAGGAG	AGTCCGCACCTTTCCCTAC	435
4	TCTGGCGGATTTCTGGGAC	CACCCATCTTCCATTCCTGC	550
5	GCCCAGAACAGCACCTAGTA	ACCAGGACCTCACAGACTTG	521
6	AGCCAGGAATGGACCATAGG	ATTGCCTCTACTGGACTGGG	393
7	GCAAGTCAGAACAAGGCCTT	GCTTTCTACCCGTGAGACCT	379
8	TACACTCAGGGACAGCTGTG	ACAGTGGGGTCCTCTCTTTG	399
9	ATCTCCCCATTTCACAGGCA	CTGCACTAGGAGGGGCTTC	329
10-1	TCAGGAGAGAGGTGAGAGCT	CTGATACAGTGGGGCAGACT	434
10-2	CCAAACTACTGTGGCCCAAG	TTTTCGGGGATCTCTGGCAT	421
10-3	AAGACTGTGAGTTCTGGGGC	TGGCAGTGATGGAACCCTAG	344
11	TCAACCTTTGCCCTCCTACT	TAAGGTGTGGCTGTCTTGGA	402

**Table 3:** Ophthalmic abnormalities of nine patients with autosomal recessive bestrophinopathy.

Patient	Age (Years)	BCVA (OD/OS)	Fundus Findings (OU)	FFA (OU)	FAF (OU)	OCT Findings (OU)	*BEST1* Variants	
							Allele 1	Allele 2
P1	79.6	20/40; 20/125	Multiple yellow subretinal deposits in the macula and around the optic disc. Serous retinal detachment. RPE alteration.	Diffuse hyperfluorescence at the macula. Descending tract pattern of hyperautofluorescence.	Hyperautofluorescence corresponding to the yellow deposits. Mixed areas of hyper and hypoautofluorescence due to RPE alterations.	Retinal thickening and fluid. Disruption of photoreceptor elongation. Disruption in the epiretinal membrane.	c.223C>T p.(L75F)	c.223C>T p.(L75F)
P2	33.8	20/40; 20/50	Yellow subretinal deposits in the macular and retinal area above the optic disc.	Diffuse hyperfluorescence at the macula.	Hyperautofluorescence corresponding to the yellow deposits. Hypoautofluorescence due to RPE alteration.	Subretinal fluid. Subretinal deposition of lipofuscin.	c.584C>T p.A195V	c.598C>T p.(R200*)
P3	34.8	20/100; 20/125	Yellow subretinal deposits in the macular and retinal area above the optic disc.	Hyperfluores-cence in the macula at the late phase.	Hyperautofluorescence corresponding to the yellow deposits. Hypoautofluorescence due to RPE alteration.	Subretinal fluid. Subretinal deposition of lipofuscin.	c.584C>T p.A195V	c.598C>T p.(R200*)
P4	32.5	20/25; 20/40	Diffuse RPE changes. Diffuse subretinal deposits at the macula and around the optic discs.	Hyperfluorescence due to RPE thinning and subretinal deposits.	Diffuse hyperautofluorescence at the macula and around the optic discs.	Subretinal fluid and diffuse deposits. Retinoschisis. RPE thinning.	c.584C>T p.A195V	c.598C>T p.(R200*)
P5	45.4	20/50; 20/50	Diffuse subretinal deposits with some foci of deposits near the vascular arcade and above the optic disc.	Diffuse hyperfluorescence at the macula.	Hyperautofluorescence at the macular. Hyporautofluorescence in the centre in a ring pattern.	Subretinal fluid and deposits. Outer retinal thinning. Photoreceptor disruption and elongation.	c.584C>T p.A195V	c.598C>T p.(R200*)
P6	48.1	20/25; 20/32	Yellow subretinal deposits at the macula. RPE alteration.	Diffuse hyperautofluorescence in the macular region.	Hyperautofluorescence at the macular. Hyporautofluorescence in the centre in a ring pattern.	Subretinal fluid and deposits. Outer retinal thinning. Photoreceptor disruption and elongation.	c.584C>T p.A195V	c.763C>T p.(R255W)
P7	42.2	20/100; 20/125	Diffuse subretinal deposits. Foci of deposits around the optic discs. Peripheral and inferior retinal alteration.	Widespread hyperautofluorescence at the macular region.	Diffuse hyperautofluorescence. Descending streaks of hyperautofluorescence.	Subretinal fluid. Outer retinal thinning.	c.598C>T p.(R200*)	c.598C>T p.(R200*)
P8	16.5	20/25; 20/32	Diffuse subretinal deposits. Foci of deposits around the optic discs and retinal vascular arcade.	Diffuse hyperluorescence. Gravitational hyperfluorescence streak.	Hyperautofluorescence at the macula and around the optic discs. Foci of hyperautofluorescence.	Subretinal fluid and deposits. Retinoschisis. Photoreceptor elongation and disruption.	c.598C>T p.(R200*)	c.865A>T p.(K289*)
P9	14.1	20/20; 20/20	Diffuse subretinal deposits. Foci of deposits around the optic discs and retinal vascular arcade.	Diffuse hyperluorescence. Gravitational hyperfluorescence streak.	Hyperautofluorescence at macula and around the optic discs. Foci of hyperautofluorescence.	Subretinal fluid and deposits. Retinoschisis. Photoreceptor elongation and disruption.	c.598C>T p.(R200*)	c.865A>T p.(K289*)

BCVA, Best Corrected Visual Acuity; OD, oculus dexter (right eye); OS, oculus sinister (left eye); FFA, fundus fluorescein angiography; FAF, fundus autofluorescence; OCT, optical coherence tomography; OU, oculus uterque (both eyes); RPE, retinal pigment epithelium.

**Table 4:** In silico analyses of five *BEST1* variants.

Exon	c.DNA Change	Amino Acid Change	MutationTaster2021	CADD v1.7	PolyPhen_2	SIFT	GnomAD v4.1.0	dbSNP157	ClinVar	Literature	ACMG Classification
3	c.223C>T	p.(L75F)	D	D	B	T	2/1614224	rs1335203485	3767340 LP	[43]	LP (PM1, PM2, PM5, PP3 and PP5)
5	c.584C>T	p.(A195V)	D	D	D	D	254/1614092	rs200277476	99725 P	[18,23]	P (PS3, PM1, PM2, PP1, and PP3)
5	c.598C>T	p.(R200*)	D	D			10/1613960	rs121918286	2741 P	[14,44]	P (PVS1, PM2, PP1, PP3, and PP5)
7	c.763C>T	p.(R255W)	D	D	D	D	44/1614060	rs372989281	143127 P	[27,45]	LP (PM1, PM2, PP3, and PP5)
7	c.865A>T	p.(K289*)	D	D			0	0	0	0	P (PVS1, PM2, PP1, and PP3)

D, damaging or deleterious; B, benign; T, tolerated; P, pathogenic; LP, likely pathogenic; PVS, pathogenic very strong; PS, pathogenic strong; PM, pathogenic moderate; PP, pathogenic supporting.

## Data Availability

The original contributions presented in this study are included in the article. Further inquiries can be directed to the corresponding authors.

## Abbreviations

The following abbreviations are used in this manuscript:

ARB	Autosomal recessive bestrophinopathy
CaCC	Calcium-activated chloride channel
BVMD	Best vitelliform macular dystrophy
EOG	Electrooculography
FAF	Fundus autofluorescence
FFA	Fundus fluorescein angiography
OCT	Optical coherence tomography
PCR	Polymerase chain reaction
gnomAD	Genome Aggregation Database
CADD	Combined annotation dependent depletion
SIFT	Sorting intolerant from tolerant
PolyPhen_2	Polymorphism phenotyping v2
ACMG	American College of Medical Genetics and Genomics
BCVA	Best corrected visual acuity
OD	Oculus dexter (right eye)
OS	Oculus sinister (left eye)
RPE	Retinal pigment epithelium
